# Potential of Lactoferrin in the Treatment of Lung Diseases

**DOI:** 10.3390/ph16020192

**Published:** 2023-01-28

**Authors:** Katarzyna Kaczyńska, Monika Jampolska, Piotr Wojciechowski, Dorota Sulejczak, Kryspin Andrzejewski, Dominika Zając

**Affiliations:** 1 Department of Respiration Physiology, Mossakowski Medical Research Institute, Polish Academy of Sciences, Pawińskiego 5 St., 02-106 Warsaw, Poland; 2 Department of Experimental Pharmacology, Mossakowski Medical Research Institute, Polish Academy of Sciences, Pawińskiego 5 St., 02-106 Warsaw, Poland

**Keywords:** lactoferrin, lung diseases, respiratory infectious diseases, asthma, lung cancer

## Abstract

Lactoferrin (LF) is a multifunctional iron-binding glycoprotein that exhibits a variety of properties, such as immunomodulatory, anti-inflammatory, antimicrobial, and anticancer, that can be used to treat numerous diseases. Lung diseases continue to be the leading cause of death and disability worldwide. Many of the therapies currently used to treat these diseases have limited efficacy or are associated with side effects. Therefore, there is a constant pursuit for new drugs and therapies, and LF is frequently considered a therapeutic agent and/or adjunct to drug-based therapies for the treatment of lung diseases. This article focuses on a review of the existing and most up-to-date literature on the contribution of the beneficial effects of LF on the treatment of lung diseases, including asthma, viral infections, cystic fibrosis, or lung cancer, among others. Although in vitro and in vivo studies indicate significant potency of LF in the treatment of the listed diseases, only in the case of respiratory tract infections do human studies seem to confirm them by demonstrating the effectiveness of LF in reducing episodes of illness and shortening the recovery period. For lung cancer, COVID-19 and sepsis, the reports are conflicting, and for other diseases, there is a paucity of human studies conclusively confirming the beneficial effects of LF.

## 1. Introduction

Lung diseases remain a leading cause of death and disability worldwide [1,2]. Three respiratory diseases, such as chronic obstructive pulmonary disease (COPD), lung cancer, and asthma, have perpetuated their place among the top ten causes of death worldwide [3]. The number of people hospitalized for respiratory failure is increasing every year. According to forecasts, the incidence and number of deaths from lung disease in the global population will increase, not only due to the burden of lifestyle but also to air pollution, widespread globalization, and the threat of pandemics such as COVID-19 [1,4]. Many of the existing therapies used to treat respiratory diseases have limited effectiveness or are associated with side effects (Table 1), so there is a constant and ongoing search for new therapies. Lactoferrin (LF), also known as lactotransferrin (LTF), is an endogenous pleiotropic molecule, abundant in airway secretions, that together with other antimicrobial peptides [5] is the first line of defense responsible for antimicrobial activity against airborne pathogens [6,7]. In addition to its antibacterial and antiviral properties, LF also exhibits antioxidant, anti-inflammatory, antitumoral and immunomodulatory properties [8,9,10] thanks to which it can be considered for the treatment of various lung diseases both as a main and ancillary drug. Lactoferrin has attracted a tremendous amount of interest in recent years in the context of therapeutic applications for the treatment of a variety of health problems, and many articles have been published on the subject, including review articles [9,11,12]. Review or meta-analysis articles on lung diseases and LF are scarce, and the latter mainly deals with respiratory tract infections [13,14]. Therefore, the purpose of this review paper was to collect and describe the existing and recent literature on the role of lactoferrin in a possible wide range of lung diseases starting from asthma, COPD, and lung cancer to acute respiratory distress syndrome (ARDS), and many others.

## 2. Lactoferrin Characteristics and Its Properties

LF is a well-conserved cationic glycoprotein of the transferrin family, produced by exocrine glands and present in mammalian milk, saliva, tears, intestine and airway secretion, and secondary granules of neutrophils. LF is a monomeric polypeptide chain consisting of 692 amino acids structured into two homologous lobes (C and N), each with the ability to chelate with high affinity to a single Fe3^+^ ion [36]. Both human (hLF) and bovine (bLF) lactoferrins are highly glycosylated with *N*-glycans, which heterogeneity and complexity is believed to play an important role in protein’s biological function. Three potential glycosylation sites have been found in hLF and five in bLF [37].

One of the most important properties of lactoferrin is the mediation of the chelation process, which reduces iron overload, potentially harmful because iron may donate electrons to oxygen, leading to the formation of reactive oxygen species (ROS), such as superoxide anions and hydroxyl radicals [38]. Through binding to iron, LF simultaneously reduces its availability to pathogens that depend on it for their own growth [9,39]. Another antioxidative effect described for LF is its potential to antagonize the phenomenon known as oxygen explosion in neutrophils, causing the production of large amounts of free radicals that damage cells and tissues [40].

LF belongs to the innate immune system and is ingested with breast milk along with IgA, which is responsible for an important part of an infant’s immunity [41]. Produced by epithelial cells of most body organs and present in numerous exocrine secretions and neutrophils, it exhibits anti-inflammatory and immunomodulatory effects by affecting the function of immune system cells such as macrophages, dendritic cells, B cells and T lymphocytes, and the production of various cytokines [42,43,44,45]. LF inhibits at the level of immune cells the production and release of pro-inflammatory agents like TNF-α and IL-6 parallel to the promotion of the anti-inflammatory ones like IL-10 or TGF-β by inhibition or activation of the NF-κB signaling pathway [46].

One of the more well-known properties of LF is its activity against microorganisms. First, the already discussed iron binding by LF deprives bacteria of a vital nutrient, second LF can interact directly with the microorganisms and for bacteria, enhance phagocytosis, inhibit biofilm formation and LPS-mediated activation, and modify interactions of microbes with host cells, and in the case of fungi *Candida albicans* induce apoptosis [44,47]. The antiviral effect of LF manifests itself by counteracting virus-induced cell apoptosis and preventing the penetration of the virus into cells by binding to viral envelope proteins or viral receptors on cells [47,48].

Lactoferrin’s multifunctionality also manifests itself in its anticancer properties, indirectly through its antioxidant and anti-inflammatory effects that prevent DNA damage and subsequent tumor formation, as well as by stimulating the activity of the adaptive immune response. Direct inhibition of tumor growth results from counteracting proliferation, survival, migration, metastasis, angiogenesis and acceleration of cancer cell death [9,11,49]. LF can act as a transcription factor that binds to specific DNA sequences and stimulate numerous genes involved in the cell cycle, apoptosis, cell differentiation, proliferation, inflammation, and ultimately anti-cancer activity [8,50,51,52].

In light of the properties of LF cited above, it seems that it can also be an effective therapeutic agent or supplement in the treatment of various respiratory diseases, including chronic inflammatory diseases such as asthma or COPD, in the progress of infections as present in cystic fibrosis and COPD, lung cancer, and many others. This article reviews the literature in the context of past and recent experimental and clinical studies on the influence of lactoferrin on respiratory diseases.

## 3. The Role of Lactoferrin in Lung Diseases

### 3.1. Rhinitis and Sinusitis

Allergic rhinitis develops when the body’s immune system perceives innocuous air particles as a threat, prompting the body to release histamine and other mediators that trigger an allergic reaction inside the nose and symptoms such as sneezing and blocked or runny nose. Sinus congestion and inflammation caused by allergic rhinitis can occasionally let sinusitis set in. Symptoms of sinusitis include nasal congestion, discolored nasal drainage, sinus pressure, headache, and fever. The most common types of sinusitis are due to bacterial colonization and viral infection, and these can cause irritation and inflammation, obstructing the drainage of mucus [53,54,55]. The question remains whether supplementation with LF, a multipotent molecule, can reduce the risk of rhinitis, shorten the illness or minimize the chances of complications, such as bronchitis. There is a conviction that consuming cow’s milk early in life, as well as raw milk in childhood, is associated with a lower incidence of allergies, respiratory infections and asthma [56,57]. Abundant milk proteins, such as IgA, IgG, IgM, TGF-β, IL-10, lactadherin, lysozyme, and among them, lactoferrin, may contribute to the induction of adaptive immune responses, and build a microenvironment that favors Treg cell development, modulate microbiota composition, and support the overall function of the intestinal barrier [57]. In addition, bovine milk contains the immunomodulatory TGF-β2 and IL-10 that induce Treg cells and lead to the production of allergen-neutralizing IgA and IgG4, but not IgE. Regarding LF, its beneficial properties were confirmed in infants receiving LF-enriched formula, who showed a lower rate of respiratory-related illnesses and fewer symptoms of runny nose, cough and wheezing [58,59,60]. Furthermore, LF may play a role in controlling polyp formation, as native and human milk-derived LF was able to inhibit nasal fibroblast proliferation [61].

#### 3.1.1. Allergic Rhinitis

LF, produced by serous cells of the submucosal glands, is present in nasal secretions [62], where its concentration increases rapidly after provocation with methacholine, histamine, or allergen [63,64]. It was suggested that LF could be a biomarker for the early detection of allergic rhinitis, as serum LF concentration combined with antigen-specific IgE levels predicted allergic rhinitis with a sensitivity and specificity of 76% and 79%, respectively [65]. An experimental study in mice model of allergic rhinitis gives hope for the therapeutic potential of LF. Intranasally applied recombinant human LF (rhLF) had a better anti-inflammatory outcome before, rather than after, the ovalbumin (OVA) challenge and restrained allergic inflammation in the mice by promoting endogenous LF expression and skewing T cells to a Th1 phenotype in the nasal mucosa [66].

#### 3.1.2. Rhinosinusitis—Viral Infection

The effect of LF on viruses that also cause sinusitis has been reported in several studies [67]. The bLF has been recently shown to prevent rhinovirus B-14 adhesion and entry into H1-HeLa cells [68]. Rhinoviruses, seasonal pathogens, and major causes of the common cold are also associated with the exacerbation of asthma. Human milk LF attenuated respiratory syncytial virus (RSV) growth and decreased its infectivity in HEp-2 cells [69,70], which was not replicated with bLF in the mouse RSV infection model [71]. The antiviral activities of bLF against enterovirus 71 (EV71) have been reported and demonstrated that LF binds to the VP1 protein of the virus and protects human cell lines against infection. LF also induced IFN-α expression of SK-N-SH cells and inhibited EV71-induced IL-6 production. Furthermore, bLF was able to protect mice against the lethal EV71 challenge [72]. The anti-adenoviral effect of LF and its N-terminal peptide lactoferricin has been shown to occur when the virus attaches to the cell membrane, mainly through competition for common glycosaminoglycan receptors [73].

#### 3.1.3. Bacterial Rhinosinusitis

The antimicrobial potential of LF against the bacteria most commonly isolated in chronic rhinosinusitis [74] has been reported. It has been shown that iron-free LF, apolactoferrin (apo-LF), binds to a common colonizer of the human nasopharynx, *Streptococcus pneumonia,* via its surface protein A (PspA) and concomitant addition of apo-LF to lytic lysozyme evokes synergistic pneumococcal killing [75]. Human milk LF could reduce the pathogenic potential of *Haemophilus influenzae* by selectively cleaving the IgA1 protease preprotein from the bacteria’s outer membrane and degrading the Hap adhesin, thus preventing Hap-mediated adherence and perturbing bacterial colonization [76]. In addition, the bacteriostatic effect of LF on *Staphylococcus aureus* growth, depending on its ferrochelating properties, has been demonstrated [77].

### 3.2. Infectious Respiratory Diseases

#### 3.2.1. Influenza

LF acts not only on viruses pathogenic to the sinuses but also on the influenza virus. The influenza virus infects the upper respiratory tract of humans and induces a variety of symptoms, such as nasal secretions, cough, headache and fever [78,79]. LF presents several antimicrobial actions by preventing the fusion of viruses into host cell proteins, counteracting viral assembly, and increasing the immune response of the host. The first occurs in the early phase of infection when LF blocks the fusion of the viral envelope protein hemagglutinin (HA) with the sialylated glycan receptor on the membrane of the host epithelial cell [7,80]. LF binds not only to the viral hemagglutinin protein but also to the host receptor molecules. Therefore, LF plays the role of an excess analog of the viral binding site, thereby increasing the competition between cellular receptors and the virus, which limits the infection [80,81]. It seems that LF glycosylation may play a role in that process [37] as sialylated oligosaccharides can act as influenza hemagglutinin blockers against influenza viruses in vivo and in vitro [82]. The dissection of LF in the C- and N lobes resulted in the discovery that the C lobe was responsible for the inhibition of the influenza virus (subtypes H1N1 and H3N2). The C lobe of bLF strongly and precisely binds to the highly conserved region containing fusion peptides of the HA2 region of viral hemagglutinin. This led to the discovery of three potent tetrapeptides (Ac-SKHS-NH_2_, Ac-SLDC-NH_2_, and Ac-SAHS-NH_2_) binding to hemagglutinin and preventing cell infection with high efficacy at fentomolar concentration [82]. Those small sequences of a broad spectrum of activity are thought as a starting point to design and develop new anti-influenza therapeutics [83]. LF not only acts as a respiratory antiviral agent by preventing the adhesion step to the host cell [84]. It also upregulates the antiviral response of the immune system. Administration of LF enhances Th1 cytokine responses and activity of immune cells such as NK-cells, monocyte/macrophages and granulocytes, which play an important role during the early phases of viral infection before the specific immune system takes over the antiviral response [85]. Downstream of the infection process, LF, by interfering with the function of caspase-3, the main effector of virus-induced apoptosis, inhibits programmed cell death and, by blocking nuclear export of viral ribonucleoproteins, prevents viral assembly. All of the activities were independent of LF iron saturation, sialylation, or glycosylation levels [86]. The effectiveness of bLF was shown in mice infected with the influenza virus, where its oral administration reduced the lung consolidation score, the number of infiltrating leukocytes in bronchoalveolar lavage fluid (BALF), and inhibited the hyperreaction of the host immune system through attenuation of pneumonia [87,88]. bLF was also shown to be as effective adjuvant as aluminum hydrogel but safer when immunizing neonatal mice against the H1N1 influenza virus [89]. A meta-analysis of randomized controlled trials by Ali et al. [13] summarized the effect of LF on the risk of respiratory infections and showed a significantly reduced likelihood of developing respiratory infections after LF use, suggesting its utility in preventing and treating various respiratory infections, including influenza. Unfortunately, the studies included in this meta-analysis were not very large in the number of patients studied, and some did not identify the cause of the respiratory tract infection. Nevertheless, there was also an earlier prospective, randomized, double-blind, placebo-controlled study carried out on healthy volunteers over three different winter seasons, which also presented significant clinical benefits of LF on the incidence and severity of respiratory tract infections, including flu, during the cold season [90].

#### 3.2.2. COVID-19

Coronavirus disease 2019 (COVID-19) is an acute respiratory infectious disease caused by SARS-CoV-2 virus infection, a highly pathogenic single-stranded (positive) RNA virus belonging to the β-coronavirus family responsible for the COVID-19 pandemic [91,92,93]. It mainly infects a person’s respiratory tract, causing fever, fatigue, headache and muscle aches, dry cough, and shortness of breath. Immunocompromised patients can develop serious complications resulting even in death. These include, for example, respiratory distress syndrome (ARDS), sepsis, metabolic acidosis, or blood clotting disorders.

The SARS-CoV-2 spike protein binds to heparan sulfate proteoglycans, attaching the virus to the surface of the target cell and interacts with the angiotensin-converting enzyme 2 receptor (ACE2), allowing the virus to enter the cell [94,95].

**Figure 1:**
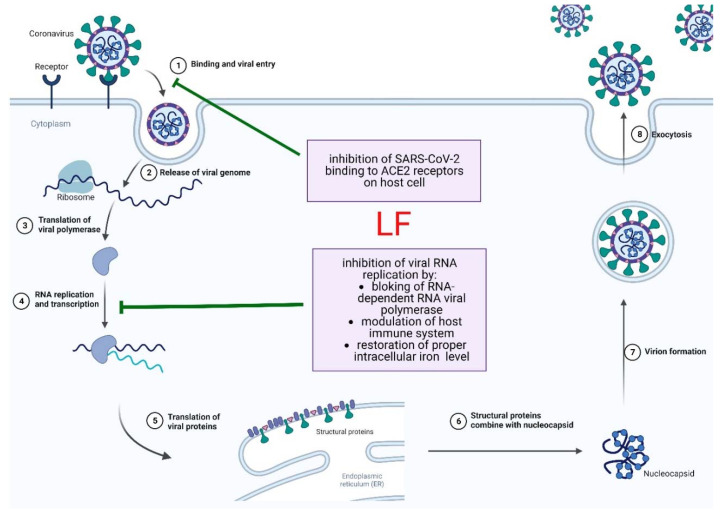
Direct and indirect action of LF on SARS-CoV-2. Adapted from “Coronavirus Replication Cycle” by BioRender.com (2020). Retrieved from https://app.biorender.com/biorender-templates accessed on 29 December 2022.

The virus then releases RNA into the cell cytoplasm, inducing the production of early viral proteins, the replication of genetic material and the production of late viral proteins, followed by the assembly of progeny virions and their release by exocytosis [96,97]. Recent data indicate the antiviral potential of human and cow’s milk directed against SARS-CoV-2 and similar viruses [98,99]. For example, although viral RNA was confirmed in the milk of mothers infected with the SARS-CoV-2 virus, no infectious viral particles were isolated from it [100]. In particular, the dual beneficial effects of bovine LF have been demonstrated during SARS-CoV-2 infection. First, LF blocks the ability of the virus spike protein to bind to the ACE2 receptor, preventing the virus from entering the target cells. In addition, it inhibits viral RNA-dependent polymerase, preventing the formation of progeny viral particles. It is known that this polymerase, as well as a helicase and 3CL proteases, are highly conserved proteins that are essential for the normal replication cycle of coronaviruses [99]. The importance of LF as an immunomodulator during COVID-19 is also highlighted, and it has recently been shown that LF causes an increase in the expression levels of genes related to the cell’s immune response to viral infection, which can interrupt SARS-CoV-2 infection [101,102]. The anti-SARS-CoV-2 effect of LF may also be related to a decrease in intracellular iron levels leading to impaired Sars-CoV-2 virus replication [103,104]. It is known that high intracellular iron levels can increase the replication of some viruses [105]. Figure 1 shows the LF targets during the replication cycle of SARS-CoV-2.

Several clinical trials have confirmed that intranasal as well as oral administration of bovine LF improves the condition of patients with COVID-19, most probably via inhibition of the SARS-CoV-2 virus [106,107,108]. Of course, these studies are small in number and preliminary, but they indicate the effectiveness and potential of LF in COVID-19 therapy. Therefore, there is a need for more high-quality and large in number human studies confirming the latter results.

### 3.3. Asthma

Asthma is a heterogeneous inflammatory disease of the airways characterized by reversible airflow obstruction, hyperreactivity of the airways, increased inflammatory cell infiltration and mucus production. Inflammation is accompanied by increased oxidative stress, which is impossible to be balanced with natural antioxidative mechanisms. In the later stages of the disease, airway remodeling is observed as a consequence of impaired collagen disposition and hypertrophy of the airway smooth muscle. Risk factors for the development and progression of asthma include air pollution, genetic and environmental factors, prenatal and early childhood exposure to certain medications, and obesity [22,109,110]. More recently, impairment of the natural intestinal microbiota, which is highly influenced by lactoferrin, has also been considered a risk factor for immune-related diseases [111]. The immunomodulatory effect of LF, described by various authors [10,112], could be beneficial in the treatment of asthma. Higher levels of LF are observed in BALF and epithelial lining fluid of adult nonsmoking asthmatics compared to healthy controls, which may be related to the permanent activation of airway epithelial cells in asthma [113]. The same has been observed post-mortem in pulmonary tissue after fatal exacerbations of asthma [114]. In general, a higher level of LF is observed during inflammation [44], where synthesis and release of LF in epithelial cells is activated by allergen challenge or pro-inflammatory agents, and in addition, LF can be released by neutrophils [115,116,117,118]. The increase in neutrophil LF release correlates with the IgE levels to the respective allergen in atopic subjects and with the severity of asthma and seems to be time- and dose-dependent in relation to allergen exposure [115].

The anti-inflammatory action of LF is based on the modulation of cytokine and chemokine production, inhibition of reactive oxygen species (ROS) generation, and reduction of immune cell recruitment [44]. Its anti-inflammatory properties have been described in mice sensitized with ragweed extract, where a single dose of LF reduced inflammatory cell accumulation, oxidative stress markers, and mucus production together with airway hyperreactivity (AHR). Given before sensitization, LF prevented inflammation, while given during it reduced the process [119,120]. In a similar vein, a recent study found that in an ovalbumin (OVA) mice model of asthma, oral LF during sensitization abolished airway hyperreactivity and inflammation by decreasing the elevated levels of TNF-α, IL-4, IL-5, IL-13 and increasing the downregulated levels of the anti-inflammatory IL-10 in BALF [121].

LF showed anti-asthmatic properties independently of species, and the asthma model since human recombinant LF (hrLF) diminished the main asthma symptoms, like AHR, and the cellular influx in the house dust mite (HDM) model introduced on non-human primates [122]. However, LF can also be an inducer of asthma-related symptoms, including eosinophilia, goblet cell hyperplasia, increased collagen deposition in the airways, and AHR, as it has been displayed for human LF in mice [123]. Shinagawa et al. (2020) [124] indicated that bLF induced a case of occupational asthma and AHR in a susceptible human subject; after inhalation contact with bLF powder, regardless of cow’s milk allergy. This points to the importance of differences in the structure of LF. However, oral preparations of bovine LF are believed to be safe and non-allergenic.

Perhaps the most important action of LF in asthma is its ability to reduce oxidative stress. This occurs at two levels. First, LF binds to free iron and thus prevents its involvement in the generation of oxidative stress and its markers [125]. As a consequence, a decrease in levels of inflammatory cytokine production and release is observed [120,125], together with a correction of the oxidative-stress-related imbalance between Th1 and Th2 responses [112]. It seems that LF controls the oxidant–antioxidant balance by sequestration of iron; however, the inhibition of free radical production also occurs in the absence of iron, up to now unknown, independent manner [125]. The sequestration of free iron further reduces lipid peroxidation and, in general, oxidation of other biologically active macromolecules and therefore protects against stress-induced oxidative damage [10]. Second, LF up-regulates the synthesis of antioxidant enzymes such as SOD [10]. LF can also be regarded as an inflammatory cell mediator that bridges various immune functions. LF inhibits the eotaxin- and GM-CSF-stimulated eosinophil migration, which seems to be independent of its iron-binding activity [126]. Moreover, LF inhibits neutrophil chemotaxis via modulation of signaling pathways responsible for neutrophil adhesion and motility, such as the calmodulin pathway [127]. Another feature of LF is its ability to push the impaired asthma balance of Th1/Th2 responses toward the Th1 [44,128]. One of the possible mechanisms of this phenomenon is its ability to suppress the Th2 polarizing chemokine CCL17 and decrease the number of Th2 and Th17 cells [129]. The immunomodulatory effects of LF are related to the inhibitory activity towards proteases, including those released by activated mast cells, such as tryptase and cathepsin G. The activation of mast cells is a key event of allergic inflammation, and its inhibition by LF, being able to enter into mast cells and thus, acting on-site, limits the degree of protease-induced inflammation and airway remodeling [130,131]. At the same time, LF does not inhibit the release of histamine from lung mast cells. In the case of cutaneous symptoms of allergy, LF stabilized the mast cells to a higher degree than most marketed anti-allergic drugs [131].

Despite the promising properties of LF towards asthma features, new data indicating its use in asthma treatment are missing. Only a few reports on the influence of LF on asthma have been published in the last ten years. All of them dealt with animal or in vitro studies; only one paper described LF-induced asthma in humans [124]. There is a patent application for LF use in asthma [132] and the phase II Clinical Trial [133] but without any further promising results. LF is used worldwide as an immunomodulator in the prevention of respiratory diseases as an OTC dietary supplement [13]; however, its use in asthma treatment, even as an additional therapy, is not yet established.

#### Breastfeeding and/or Lactoferrin-Enriched Formulae and Risk Factors for Developing Asthma in the Childhood

WHO recommends exclusive breastfeeding up to the age of six months and, accompanied by complementary food, for up to two years (WHO). It is generally accepted that breastfeeding enhances the child’s immunity and diminishes the risk of developing asthma [134,135,136,137,138]. Exclusive breastfeeding during the first six months of life reduces the risk of developing asthma by up to two years and any longer by up to five years [139]. This period of protection maybe even longer and encompasses even the entire childhood, especially in low- and middle-income counties [140,141]. In contrast, some researchers could not find in high-income countries a relationship between breastfeeding and the risk of developing asthma, which may be difficult to establish due to the extensive use of modified milk/formula feeding [142,143]. There are also studies showing that the consumption of raw cow’s milk by children in Poland and Germany reduced the incidence of asthma [56,144]. These observations have been confirmed in a HDM-induced murine model of asthma where administration of raw cow milk during sensitization prevented the development of allergic airway inflammation and decreased levels of Th2 cytokines, together with the cellular influx into the airways [129]. Thus, the beneficial action of breastfeeding or raw milk consumption on asthma development appears to rely on the enhancement of the proper development of the child’s gut microbiota, providing live microbes and oligosaccharides and pre- and probiotics. Second, milk contains various immunomodulators, anti-bacterial, antiviral and anti-inflammatory agents, including lactoferrin, as well as pro- and anti-inflammatory cytokines, including IL-4, 5, 13, 10, TGF-β, secretory IgA and long-chain fatty acids [145,146]. Therefore, it may protect against respiratory infections in early life, which are one of the risk factors for developing asthma.

### 3.4. Chronic Obstructive Pulmonary Disease

Chronic obstructive pulmonary disease (COPD) is a widely prevalent debilitating inflammatory lung disease, estimated to affect more than 380 million people and is the third leading cause of death in the world [147,148]. COPD is defined by irreversible airflow obstruction with persistent respiratory symptoms such as obstructive bronchiolitis, dyspnea, cough, emphysema, and exorbitant sputum production [149]. The predominant risk factor for developing COPD is smoking and air pollution. The progression and exacerbations often are the result of bacterial infections [150,151]. Bronchial epithelium and bronchoalveolar lavage contain numerous antibacterial proteins and peptides, among them lactoferrin [150,152,153], which acts as an iron chelator to inhibit bacterial growth and biofilm formation [154,155].

**Figure 2:**
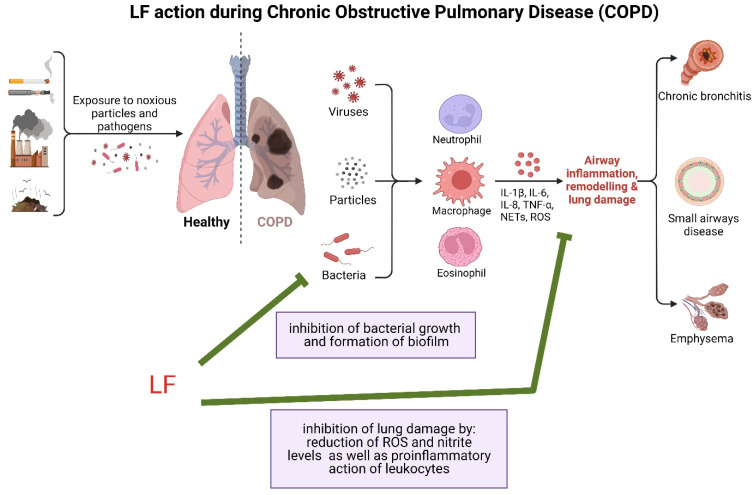
Potential beneficial effects of LF in the course of COPD. Adapted from “Development of Chronic Obstructive Pulmonary Disease (COPD)”, by BioRender.com (2020). Retrieved from https://app.biorender.com/biorender-templates accessed on 2 January 2023.

Goblet cell and submucosal gland hyperplasia and airway secretory capacity are increased in COPD [150,152]. Consequently, elevated LF levels are found in COPD patients compared to healthy controls [151,152,156]. BALF samples from smokers had a higher iron-to-lactoferrin ratio, and supplementation of BALF collected from smokers with LF resulted in a concentration-dependent reduction in bacterial growth and biofilm formation [155]. COPD is an oxidative stress-based pathology; therefore, antioxidant LF activity could be of great importance in therapy. In fact, bLF aerosol treatment significantly reduced oxidative lung injury in hyperoxic mice [157]. The combination of LF with the medicinal plant *Pelargonium sidoides* decreased the level of reactive oxygen species (ROS), and nitrite in macrophagic cells stimulated with LPS [158]. With its antimicrobial and antioxidant activity, LF appears to be a good candidate for COPD therapy (Figure 2), but more preclinical studies in COPD models are needed to prove its efficacy.

### 3.5. Cystic Fibrosis

Cystic fibrosis (CF) is a genetic disease caused by the presence of mutations in both copies of the gene for the cystic fibrosis transmembrane conductance regulator (CFTR) protein, which affects primarily the respiratory system and the gastrointestinal tract. In the case of the respiratory tract, non-functional CFTR responds to thick mucus that clogs the bronchi and bronchioles, promotes infections caused by bacteria, and causes chronic inflammation leading to lung damage and respiratory failure [159]. It appears that LF, due to its antibacterial and anti-inflammatory activity, could have a supportive effect on limiting bacterial growth, reducing neutrophil recruitment, and pro-inflammatory cytokines in cystic fibrosis [160]. The level of LF in BALF and saliva of CF patients is elevated [161,162], but not its microbicidal activity [163]. In a murine model of chronic lung infection induced by *Pseudomonas aeruginosa*, aerosolized bLF was able to reduce neutrophil recruitment and levels of pro-inflammatory interleukins and chemokines [164]. Similarly, in the CF model in mice deficient in CFTR and infected with *P. aeruginosa*, aerosolized bLF restricted infection by reducing pulmonary bacterial load, inflammation and iron dysbalance [165]. An in vitro study in the A549 human bronchial cell line showed an anti-invasive effect of bLF on *Pseudomonas aeruginosa* and *Burkholderia cenocepacia*, two important opportunistic respiratory pathogens in patients with CF [166]. Bovine LF applied to the bronchial epithelium infected with *P. aeruginosa*, all isolated from CF patients, reduced intracellular bacterial survival and inflammatory response by reducing IL-1β, IL-6, and IL-8 levels [167]. Furthermore, LF combined with hypothiocyanin intensified the ability of tobramycin and aztreonam to eliminate *P. aeruginosa* biofilms growing in airway epithelial cells [168] and was able to effectively kill this pathogen in the sputum of CF patients with greater activity than tobramycin [169]. Overall, these data provide strong encouragement for the idea of using LF to treat CF.

### 3.6. Acute Respiratory Distress Syndrome

Acute lung injury (ALI) and its rapidly progressing form—acute respiratory distress syndrome (ARDS)—is a severe form of acute hypoxemic respiratory failure, defined by damage to the alveolar-capillary membrane, pulmonary edema, decreased lung compliance, and the need for mechanical ventilation. ARDS, burdened with a high mortality risk, also characterized by widespread lung inflammation, uncontrolled oxidative stress, and neutrophil accumulation, occurs most frequently in pneumonia, sepsis, aspiration of gastric fluids, trauma, and harmful gas inhalation [170,171].

Bovine lactoferrin has been widely described as an effective agent that relieves lung injury in experimental studies. The therapeutic effect of aerosolized bLF has been shown to decrease lung edema, cell infiltration in BALF, inflammatory cytokines (IL-1β and IL-6), general lung injury, and mortality rate in mice exposed to hyperoxia [157]. In a rat model of sepsis-induced acute lung injury, orally administered bLF counteracted inflammatory cell invasion, including neutrophilis, oxidative stress, and lung edema [172]. Likewise, human LF administered intraperitoneally, prophylactically, and therapeutically in lipopolysaccharide-induced ALI, alleviated pulmonary edema, alveolar hemorrhage, inflammatory cell infiltration, TNF-α production, and increased interleukin-10 in mouse BALF [173].

In the context of human studies, it has been noted that the BALF of ARDS patients contains increased levels of iron and LF, which may have been linked to a host response that reduces both catalytically active iron and related oxidative stress [174].

There are reports on the effect of lactoferrin in reducing the risk of sepsis, a common cause of ARDS. Several randomized control trials (RCTs) showed that supplementation of bLF reduced the incidence of late-onset sepsis in premature neonates with very low birth weight [175,176,177]. However, a meta-analysis of data from 12 RCTs showed that LF supplementation in enteral feeding of preterm infants reduces the risk of late infections rather in small studies with poor methodology and publication bias, while large studies with a good methodological quality show no evidence of an effect [178]. The Enteral Lactoferrin in Neonates (ELFIN) study from the UK, published later, also failed to show that bLF given to premature infants was effective in reducing late sepsis and necrotizing enterocolitis [179,180]. Contradictory results were also obtained in studies on adult patients suffering from acute sepsis. One of them, a randomized, double-blind, placebo-controlled phase II study on 192 patients, showed that enteral talactoferrin (recombinant human lactoferrin, TLF) significantly reduced mortality in patients with severe sepsis [181], while a phase II/III clinical trial conducted in a similar regimen on 305 patients did not confirm this, and even reported increased mortality in the TLF-treated group [182].

Much attention has recently been paid to the potency of LF in mitigating SARS-CoV- 2 infection and subsequent ARDS. In addition to its antiviral activity towards SARS-CoV-2 described in Section 3.2.2. LF presents with anti-inflammatory, anti-infective, and immune-regulating properties, which could be responsible for the reduction of inflammatory response and oxidative stress and prevention of subsequent overactive immune response, i.e., “cytokine storm” [183], and reactive oxygen species induced cell and tissue damage [8,10]. The ability of LF to activate inflammatory cells [184,185], downregulate the expression of various chemotactic factors and adhesion molecules (ICAM-1, E-selectin) [186,187] and reduce pro-inflammatory cytokine secretion such as IL-6, IL-1β and TNF-α [8,157,173,188], in in vivo and in vitro models have been well described, thus pointing to its potency in the treatment of COVID-19 induced ARDS.

However, studies in humans examining the effects of LF on SARS-CoV-2 infection and ARDS are limited, and evidence of its benefits is lacking. A randomized pilot study involving a small group of participants (54) with mild to moderate COVID-19 symptoms showed a trend toward a positive effect of LF, but differences in symptom or laboratory improvement between the control and the LF-treated groups were nonsignificant [189]. From the above articles, especially from animal studies, it appears that LF has significant potency in reducing the symptoms of lung injury in ALI/ARDS, while we lack clinical trials to confirm its efficacy.

### 3.7. Lung Cancer

The anticancer properties of lactoferrin related to immunomodulation, apoptosis induction, cell cycle modulation, and inhibition of cell migration, invasion, and metastasis have been previously reviewed for a variety of cancer types [9,11,49]. Not surprisingly, interest in such effects of LF has emerged in the context of therapy of one of the most common and with poor prognosis types of malignancies, lung cancer. Interestingly, the lactotransferrin gene, referred to as the lactoferrin gene, may play an important role in lung carcinogenesis. It is inactivated in most lung cancers by genetic or epigenetic mechanisms, and its expression can be restored in some cell lines after treatment with a demethylating agent or histone deacetylase inhibitor [190]. One of the first studies showed that both iron-free and iron-saturated human LF significantly reduced lung metastasis of B16-F10 melanoma cells in syngeneic mice [191]. This is supported by the recent study using LF knockout mice, where LF deficiency facilitated metastasizing melanoma cells in the lungs by repressing TLR9 signaling and recruiting myeloid-derived suppressor cells in the lungs [192].

Talactoferrin has been applied to treat non-small cell lung cancer (NSCLC), the most prevalent form of lung cancer that constitutes over 80% of cases [193,194]. TLF is an immunomodulatory protein that, when administered orally, activates the recruitment and maturation of dendritic cells that carry tumor antigens in gut-associated lymphoid tissue and activates the production of cytokines. All of these are likely to promote the maturation and proliferation of natural anticancer killer (NK) cells, CD8 lymphocytes and NK-T cells. Consequently, the LF-induced activation of tumor-draining lymph nodes and tumor cellular infiltration may lead to tumor cell death [195]. Phase II trials in advanced or metastatic NSCLC cancer, conducted only in India, have shown increased antitumor activity of carboplatin/paclitaxel combined with oral TLF versus carboplatin/paclitaxel alone [193] and improved overall survival when TLF was used alone [194]. Despite promising results from Phase II studies, subsequent Phase III multicenter studies involving significantly larger numbers of subjects were disappointingly negative for overall survival as well as disease progression-free survival in patients with advanced NSCLC [196].

LF has been shown to have potential use in the treatment or chemoprevention of lung adenocarcinoma, the most prevalent and aggressive subtype of NSCLC. Bovine LF appeared to inhibit lung cancer growth by inhibiting tumor-induced angiogenesis dependent on the vascular endothelial growth factor (VEGF) pathway [197]. Treatment with bLF was able to reduce the proliferation of a human lung adenocarcinoma cell line (A549) by decreasing the expression of the VEGF protein and oral bLF in transgenic mice overexpressing VEGF and developing lung tumors suppressed inflammation, eliminated the expression of hVEGF-A165 and reduced solid tumor formation [197]. In another study, bLF has been shown to form a complex with immunoglobulin (CD79A) binding protein 1 (IGBP1), which interacted with the catalytic subunit of protein phosphatase 2A (PP2A) to inhibit its activity and promote apoptosis of PC-14 lung adenocarcinoma cells [198]. Antitumor LF activity exhibited as inhibition of cell viability, migration, and reduced tumor weight has been confirmed in vitro and in vivo models with lung tumor cell line A549 through the induction of cell apoptosis [199]. This was evidenced by elevated levels of the apoptosis-increasing factors Bax and a caspase-3 and reduced level of the apoptosis inhibitor Bcl-2. The latest study, exploiting a novel recombinant human LF (rhLF), also reported suppression of the growth and migration of adenocarcinoma [200]. The mechanism of adenocarcinoma cell death was due to the induction of apoptosis, as rhLF increased the percentage of apoptotic cells, externalization of phosphatidylserine (PS), increased levels of caspase-3 activity, and cell cycle arrest in phase S in rhLF-treated cancer cells. Due to its glycosylation profile, which is consistent with natural human LF, rhLF could potentially be considered for parenteral therapeutic use in humans due to its low risk of antigenic incompatibility and its proven lack of cytotoxicity toward normal human bronchial epithelial cells [200].

LF has been used not only as an anticancer agent but also as a specific carrier, enhancing the ability to actively deliver the drug to the tumor. Natural polymers with intrinsic tumor-targeting abilities and LF in combination with chondroitin were used to functionalize the surface of nanoparticles encapsulating cytotoxic chemotherapeutic drugs. Inhalable nanocomposites proved to be much more effective in mice with their antitumor effects in lung cancer, compared to the spray-dried free drug powder mixture [201,202].

In the context of the studies cited above, LF appears to be a potential candidate for the treatment of lung cancer, whether used as monotherapy or perhaps in synergy with other conventional drugs [200], or even as a carrier of anticancer drugs, although more studies, especially clinical ones, are needed to confirm its efficacy and safety. The effects of LF on lung cancer are summarized in Table 2.

**Figure 3:**
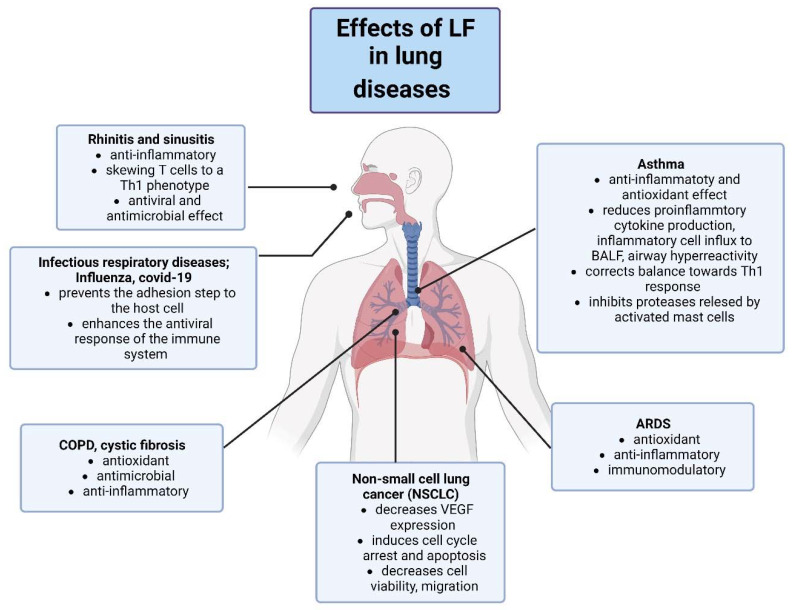
Summary of mechanisms of therapeutic action of LF in various respiratory and pulmonary diseases. COPD; chronic obstructive pulmonary disease, ARDS; acute respiratory distress syndrome. Created with BioRender.com.

Information summarizing the beneficial effects of LF in various respiratory and pulmonary diseases is presented in Figure 3.

## 4. LF Forms and Application

Most scientific research is conducted with bLF, which is regarded as safe by the Food and Drug Administration (USA) and is commercially accessible in abundance [203]. The others use human recombinant LF, which is important for translating research results into the clinic, given the low risk of antigenic incompatibility [200]. In in vivo studies, or human use, LF is most often administered orally as a non-invasive and practical route. However, it is important to remember that the bioavailability of orally administered LF varies during growth, with a higher rate of intestinal absorption among infants and less absorption during adulthood [11]. For example, gastric survival of oral bLF in adults was above 60% [204]. bLF has been used successfully in clinical trials in adults and children using bLF-enriched formulas, where it improved various health issues [205]. LF is available on the market as food products (enriched drinks, yogurt) or supplements in the form of bulk powders, capsules, and syrups, where these products are not subject to a controlled reporting system of effectiveness [108,205,206].

Less common pathways for LF application in in vivo and human studies include systemic injection [207,208] as well as noninvasive topical, intravaginal [209] or on the eye surface [210]. For the treatment of respiratory and pulmonary diseases, although not exclusively, the administration of LF in aerosol or intranasal form seems to be the most optimal. In fact, aerosolized bLF has proven effective in reducing symptoms of chronic lung injury and infection in a mouse models. Intranasally applied rhLF was also able to suppress inflammation in allergic rhinitis in mice [66]. In a human study, preliminary clinical results have shown that LF applied in the form of nasal drops or mouth aerosol improved the outcome of patients with COVID-19 [106,107,108]. Due to the instability of lactoferrin in water and the high drainage of the nasolacrimal duct, its potential efficacy can be reduced, which is why the liposomal form of LF was used in the latter work. Liposomalization or encapsulation of LF have been demonstrated to improve its availability and effect also after topical [210] and oral application [211].

## 5. Conclusions

Lung diseases are among the most common conditions that not only impair quality of life but can also lead to death. Their treatment still requires the search for new active substances and therapeutic pathways. One of the substances known and present in the body is lactoferrin, which is economically undemanding and has limited side effects, and could be used to treat and support the treatment of various pathologies affecting the respiratory system.

Among its properties that could be useful in the treatment of asthma, influenza, COPD, or even cancer, are its antimicrobial, antioxidant, anti-inflammatory, antitumoral, and immunomodulatory activities, which have been demonstrated mainly in in vitro and in vivo models. For example, the LF used in NSCLC cancer cell lines inhibited cell survival and growth and positively affected patient survival in Phase II clinical trials; however, this effect was not replicated in Phase III studies. Similarly, in the case of COPD or cystic fibrosis, although in vitro and in vivo data indicate its effective antibacterial activity, no attempt has been made to establish its effect in human studies. However, a meta-analysis of randomized controlled trials summarizing the effect of LF on the risk of respiratory infections showed a significantly reduced likelihood of developing respiratory infections. While, LF used in asthma treatment, even as an additional therapy, is not yet established. It is difficult to draw definite conclusions from the above review due to the paucity of publications on LF and the specific disease entity. There is also a lack of good-quality human studies that could contribute to the registration of LF as a drug. Paradoxically, LF is commercially available as a dietary supplement. Unfortunately, its effectiveness when used as an adjunct therapy for various diseases is not registered or controlled in any way, so it does not contribute to the growing knowledge of its benefits. LF appears to be an excellent medicine in animal studies, but human studies show its limited therapeutic potential in lung disease, being more of an immunomodulator. To consider LF as an additional or alternative treatment for various pathologies affecting the respiratory system, we need more evidence and good-quality clinical trials to demonstrate its effectiveness in humans.

## Figures and Tables

**Table 1:** Therapeutic approaches in respiratory diseases. Their advantages and disadvantages and the possible use of LF as a direct or additive therapeutic.

Disease/ Reference	Therapy	Advantages	Disadvantages	Possible Use of LF
Allergic Rhinosinusitis				
[15]	Biologics (anti-IgE, anti-interleukin agents)	Reduction of allergy as a main cause of AR	Very high costs	
[16]	Antihistamine drugs	Fast blockade of symptoms	Limited efficacy, possible sedation, interactions with other drugs	
[16]	Intranasal corticosteroids	Very high safety and efficiency, fast action	Nasal dryness, not for long- and very long-term use	+
[16]	Allergen immunotherapy	Elimination of allergy as the main cause of allergy	Costs, possible side effects, long duration of the treatment	+
Viral and Allergic Rhinosinusitis				
	Immunomodulators	Amelioration of natural defense mechanisms/function or the organism, boosting the immune system	Lack of response in some patients, not always acknowledged by clinicians	+
Bacterial Rhinosinusitis				
[17,18]	Antibiotics	Fast resolution of symptoms, eradication of pathogens, prevention of complications	Side effects, in case of overuse, resistance to antibiotics	+
[18]	Intranasal corticosteroids combined with antibiotics	Very high safety and efficiency, fast action	Nasal dryness, not for long- and very long-term use	+
Influenza				
[19]	Antivirals (oseltamivir, zanamivir, peramivir, baloxavir and others)	Shorten disease length, prevention of influenza-related compilations	Application after first symptoms, risk of viral drug resistance	
	Immunomodulation via immunomodulators	Amelioration of natural defense mechanisms/function or the organism, boosting the immune system	Lack of response in some patients, not always acknowledged by clinicians, rather used in prevention than in the acute treatment	+
[20]	Home-based treatments (bed rest, NSAIDs, sufficient hydration)	Sufficient in case of mild infection	Not applicable in case of complications or severe infections	+
COVID-19				
[21]	Corticosteroids	Reduction of mortality	Secondary infections	
[21]	IL-6 receptor antagonist antibody	Reduction of mortality	Not known	
[21]	Anticoagulants in hospitalized patients	Reduction of risk of major thrombotic events	Higher risk of major bleeding	
[21]	Non-invasive continuous positive airway pressure/ High-flow nasal oxygen	Reduction of the need of invasive ventilation	Increased aerosol generation	
[21]	JAK inhibitors	Reduction of mortality	Not known	
Asthma				
[22]	Inhaled corticosteroids (ICS)	Good management of symptoms, better asthma control, reduction of asthma exacerbations, gold standard, usually well tolerated	Non-adherence due to difficulties in administration (wrong inhalation technique)	+
[22]	LABA (long-acting beta2-agonist) recommended only in combination with inhaled corticosteroids	When used with ICS: better lung function, reduction of asthma exacerbations, prevention of asthma progression and airway remodeling	Not recommended for monotherapy due to increased risk of fatal asthma exacerbation, possible cardiac side effects like arrhythmias, palpitations, increased risk of hyperglycemia	
[23]	LAMA (long-acting inhaled muscarinic antagonist) alone or in combination	Prevention of asthma progression, better asthma control	Different responses in different age groups recommended only in severe asthma	
[22]	SABA (short-acting beta2-agonists) (only in combination with inhaled corticosteroids when ICS alone is insufficient)	Immediate relief of bronchospasm	Increased risk of poor asthma outcome when overused	
[22]	Leukotriene receptor antagonist (LTRA, e.g., montelukast)	Alternative drugs to ICS when ICS is not tolerated	Less effective than ICS, mental health-associated side effects	
[22]	Immunotherapy in case of allergic asthma	Elimination of the causes of asthma, better asthma control, lower number of exacerbations	High costs, sometimes unavailable, not all patients respond to the treatment	+
[23]	Monoclonal anti-IL-5 antibody	Reduction of number of exacerbations	High costs	
[23]	Monoclonal anti-IL-4Rα	Reduction of asthma exacerbations, better asthma control and outcomes	Reaction at injection side	
[23]	Oral corticosteroids only in severe asthma	Immediate improvement of acute asthma exacerbation, better asthma control, reduction of asthma symptoms	Cushing’s syndrome, weight gain, hyperglycemia, diabetes/metabolic syndrome, osteoporosis, insomnia, or sleep disturbances	
COPD				
[24]	Pulmonary rehabilitation	Increased exercise capacity, reduction of hospital readmission, improvement of quality of life	Not known	
[25]	Long-term home non-invasive ventilation	Reduction of hypercapnia	Decrease in quality of life, need for specialized equipment	
[26]	Acute non-invasive ventilation during exacerbation	Reduction of hospitalizations and length of hospital stay	Cannot be performed at home	
[27]	Inhaled corticosteroids (ICS)	Reduction of inflammation	Lack of response to ICS in some patients	+
[26]	Oral corticosteroids	Improvement of lung function in ambulatory patients during exacerbations, fewer hospitalizations	Cushing’s syndrome, weight gain, hyperglycemia, diabetes/metabolic syndrome, osteoporosis, insomnia, or sleep disturbances	+
[24]	Mucolytics	Reduction of the number of hospitalization and exacerbations	Not known	+
[24]	LAMA (long-acting inhaled muscarinic antagonist (e.g., Tiotropium) alone or in combination	Reduction of risk of exacerbations, better lung function	Different responses in different age-groups	
[24]	Phosphodiesterase-4 inhibitor (roflumilast)	Reduction of number of exacerbations, better lung function	Diarrhea, nausea, weight loss, psychiatric disturbances including depression, insomnia, or sleep disturbances	
[24]	Macrolide antibiotic therapy	Reduction of number of exacerbations, improvement of quality of life	Hearing decrement, risk of ventricular arrythmia, diarrhea	+
Cystic fibrosis				
[28,29]	Antibiotics	Management of bacterial colonization and infections	Risk of antibiotic resistance	+
[28,29]	NSAIDs, mostly ibuprofen, in children	Reduction of airway inflammation	At lower doses possible increase of inflammation, bleeding from the GI, but no effects of ibuprofen in adults	
[28,29]	Inhalations with hypertonic saline and dornase alpha	Decrease of viscoelasticity of mucus, elimination of mucus	Time-consuming	
[28,29]	Physiotherapy	Better lung function	Time-consuming	
ARDS/ALI				
[30]	High-flow nasal cannula (HFNC)	high oxygenation, alveolar recruitment, increased secretion clearance, reduction of dead space	Not known	
[31]	Antibiotics in case of bacterial pneumonia	Elimination of one of the causes of ALI/ARDS	Development of multi-drug resistant pathogens	+
Lung cancer				
[32]	Radiotherapy	Decrease of pain, reduction of metastasis	Possible burns due to incidental irradiation of the surrounding tissue	
[33]	Chemotherapy	Reduction of the tumor, longer survival	Fatigue, dizziness, increased risk of infections, anemia, bleedings, diarrhea, nausea, weight loss, anxiety, smell, and taste disturbances, hair loss, etc.	+
[34]	Surgery	Physical elimination of the tumor	Surgical complications	+
[35]	Immunotherapy	Longer survival, fewer side effects compared to other treatment options	High cost	+

**Table 2:** Effects of various forms of lactoferrin applied alone or in combination with other drugs on lung cancer.

Cancer/Cancer Cell Line	Study/ Number of Participants	Lactoferrin	Effect/Mechanism	Reference
non-small cell lung cancer (NSCLC)	Clinical study (phase II)/n = 110	Talactoferrin (TLF)—recombinant human LF combined with carboplatin/paclitaxel	Improved patient survival	[193]
	Clinical study (phase II)/n = 100	TLF	Improved patient survival	[194]
	Clinical study (phase III)/n = 742	TLF	No improvement	[196]
lung adenocarcinoma cell line—A549	In vitro	bLF	Reduced proliferation by a decrease in VEGF expression	[197]
		LF (iron saturated)	Inhibition of cancer cell viability, migration, and apoptosis induction	[199]
		recombinant human LF (rhLF)	Inhibition of cell growth and migration; cell cycle arrest and induction of apoptosis	[200]
		recombinant human LF (rhLF) in combination with etoposide	Repressed cancer cell growth by cell cycle arrest and induction of apoptosis Reduction by 10-fold the etoposide dose by rhLF to achieve the same anticancer effect	[200]
lung adenocarcinoma cells—PC-14	In vitro	bLF	bLF formed a complex with immunoglobulin (CD79A) binding protein 1 (IGBP1), which interacted with the catalytic subunit of protein phosphatase 2A to promote cell apoptosis	[198]

## Data Availability

Data sharing not applicable.
